# Supplementary figures and images for: Association of TNF-α (-308G/A) Gene Polymorphism with Circulating TNF-α Levels and Excessive Daytime Sleepiness in Adults with Coronary Artery Disease and Concomitant Obstructive Sleep Apnea
Source: J Clin Med. 2021 Jul 31;10(15):3413. doi: 10.3390/jcm10153413 (PMC8348542; doi:10.3390/jcm10153413)

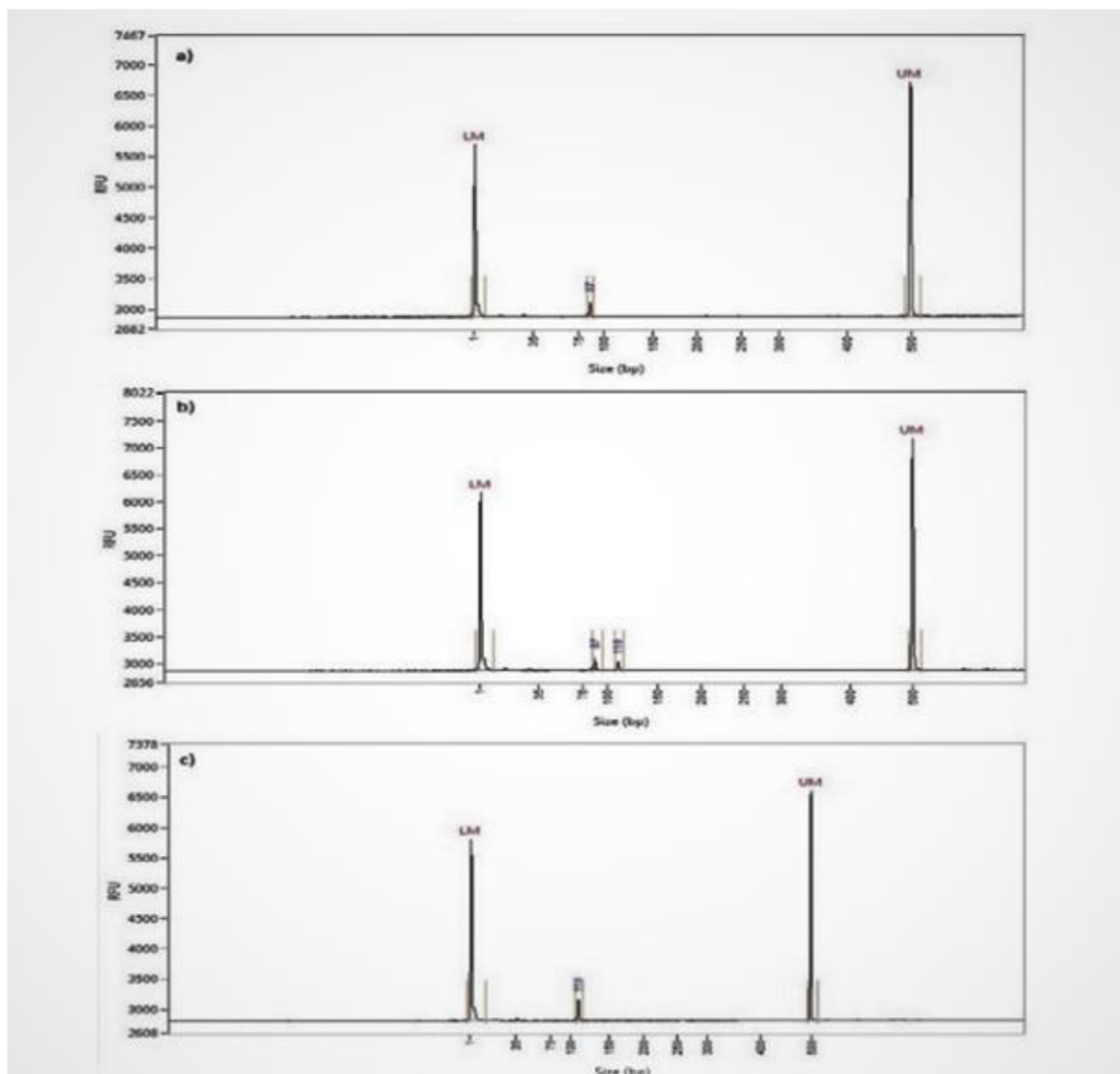

Figure S2. Sequencing for the three possible genotypes of Rs18000629 .

Supplement: Supplementary file 1 [file jcm-10-03413-s001.zip › jcm-1278431-supplementary.pdf]
